# A Pilot Study of Plantar Mechanics Distributions and Fatigue Profiles after Running on a Treadmill: Using a Support Vector Machine Algorithm

**DOI:** 10.1155/2023/7461729

**Published:** 2023-02-21

**Authors:** Qian Liu, Hairong Chen, Anand Thirupathi, Meimei Yang, Julien S. Baker, Yaodong Gu

**Affiliations:** ^1^Faculty of Sports Science, Ningbo University, Ningbo 315211, China; ^2^Department of International Office, Ningbo University, Ningbo 315211, China; ^3^CEEC Economic and Trade Cooperation Institute, Ningbo University, Ningbo 315211, China; ^4^Centre for Health and Exercise Science Research, Department of Sport, Physical Education and Health, Hong Kong Baptist University, Hong Kong 999077, China; ^5^Faculty of Engineering, University of Szeged, Szeged 6724, Hungary

## Abstract

The treadmill is widely used in running fatigue experiments, and the variation of plantar mechanical parameters caused by fatigue and gender, as well as the prediction of fatigue curves by a machine learning algorithm, play an important role in providing different training programs. This experiment aimed to compare changes in peak pressure (PP), peak force (PF), plantar impulse (PI), and gender differences of novice runners after they were fatigued by running. A support vector machine (SVM) was used to predict the fatigue curve according to the changes in PP, PF, and PI before and after fatigue. 15 healthy males and 15 healthy females completed two runs at a speed of 3.3 m/s ± 5% on a footscan pressure plate before and after fatigue. After fatigue, PP, PF, and PI decreased at hallux (*T*1) and second-fifth toes (*T*2–5), while heel medial (HM) and heel lateral (HL) increased. In addition, PP and PI also increased at the first metatarsal (*M*1). PP, PF, and PI at *T*1 and *T*2–5 were significantly higher in females than in males, and metatarsal 3–5 (*M*3–5) were significantly lower in females than in males. The SVM classification algorithm results showed the accuracy was above average level using the *T*1 PP/HL PF (train accuracy: 65%; test accuracy: 75%), *T*1 PF/HL PF (train accuracy: 67.5%; test accuracy: 65%), and HL PF/*T*1 PI (train accuracy: 67.5%; test accuracy: 70%). These values could provide information about running and gender-related injuries, such as metatarsal stress fractures and hallux valgus. Application of the SVM to the identification of plantar mechanical features before and after fatigue. The features of the plantar zones after fatigue can be identified and the learned algorithm of plantar zone combinations with above-average accuracy (*T*1 PP/HL PF, *T*1 PF/HL PF, and HL PF/*T*1 PI) can be used to predict running fatigue and supervise training. It provided an important idea for the detection of fatigue after running.

## 1. Introduction

The most serious threat to health in modern times has been identified as sedentary behavior with insufficient physical activity [1]. Running has long been a popular leisure activity. Athletes have much lower resting heart rate, body weight, body mass index (BMI), and triglyceride levels compared to the general population [2], indicating that regular physical exercise can minimize the risks of cardiovascular disease. At the same time, running carries a considerable risk of injury. In follow-up cases in the population, the incidence of running-related injury was reported to be 2.5 to 33.0 cases per 1000 h [3]. However, the causes of injuries are varied. Most running-related lower limb injuries, for example, are the result of avoidable training errors [4, 5]. In addition, accumulating long and strong training may lead to an increase in shin pain [6].

Muscle tiredness is a complicated physiological state induced not only by changes in muscle capacity but also by the central nervous system's inability to appropriately drive motor neurons [7]. Long-term running has been proven to cause central fatigue, which diminishes the strength of the maximal autonomic plantar flexor muscle. Plantar flexor fatigue can limit the power of these muscles during the propulsion phase of running, and lower limb strength can be lowered by 30 to 40% after running [8, 9]. The biomechanical features of the lower limbs change as a result of exhaustion, which is crucial in preventing sports injuries. Changes in knee angle and moment because of fatigue, for example, can be used to predict anterior cruciate ligament injuries [10].

Several measurement approaches have been utilized in many studies to quantify the association between foot dynamics and lower extremity overuse injuries. Plantar mechanical measurement has been frequently utilized to evaluate overall running performance as a result of this [11, 12]. The second and third metatarsals exhibit a 10% increase in peak pressure immediately after fatigue, and an 11% increase after 30 mins, with a significant 12% drop in load at the first toe [13]. It is worth noting that increased load under the metatarsal bone can produce biomechanical imbalance, which could lead to metatarsalgia [14]. Furthermore, the increasing plantar load will promote stretching stresses on the plantar aponeurosis, which leads to microtraumas and degradation of connective tissues, promoting the development of plantar fasciitis [15, 16]. In conclusion, there is an urgent need to reflect on and evaluate fatigue and fatigue injuries through changes in plantar mechanical parameters. Insole technologies for activity classification couple plantar pressure with accelerometer data, increasing technology cost, and complexity [17, 18]. The advantage of the platform is that it is easy to use because it is stationary and flat and can be well applied to the laboratory environment [19]. Therefore, we used the footscan force platform to detect the mechanical characteristics of the plantar. Treadmills have been widely used in laboratory studies to easily control speed gradients. Previous studies have also shown that treadmill running is different from running on the ground. Whether treadmill running can simulate running on the ground is still a controversial issue [20]. This experiment only examined the change form of plantar mechanical parameters after fatigue running on a treadmill.

Males and females have different bone structures and muscle strength, and studies have shown that females are more likely than males to sustain lower limb injuries while running [21, 22]. Females are more prone than males to have ligamentous laxity of the ankle joint, and females are approximately twice as likely as males to have ankle sprains [23]. Plantar mechanical parameter distributions are affected by several factors, including weight, gender, foot structure, and even how a person stands or walks [24]. Experts in forensic science use variations in foot bones to determine gender [25]. There are, however, no consistent results on the gender differences in plantar pressure characteristics. According to research [26], there are no significant variations in the midfoot contact area and plantar pressure between males and females. The pressure under the toes was higher in female adolescents than in male adolescents, while the pressure was higher in male adolescents only at the hindfoot, and the pressure at the metatarsophalangeal toe increased more significantly in females [27]. The difference in plantar mechanical parameters caused by gender can reflect a lot of practical problems. Therefore, it is necessary to explore the effect of gender differences on plantar mechanical parameters.

In biomechanical research, traditional statistical methodologies have limited the ability to classify groups based on many variables [28]. In recent years, a support vector machine (SVM) has emerged as a new tool for solving biological classification problems [29]. By creating discriminatory parameters to separate groups from one another, the SVM attempts to discover a hyperplane that maximizes the distance between groups [30]. The SVM has the advantage of producing classification results based on limited data sets while minimizing structural and empirical risk [31]. Injuries are common in individual sports and will cause serious physical outcomes. Reduced exercise capacity because of fatigue increases the incidence of musculoskeletal injuries [32]. As a result, forecasting the occurrence of sports injuries is critical to maintaining good health [33]. Previous research [34] used the SVM to predict diabetic foot ulceration based on plantar mechanical parameters. Aguirre et al. [35] proposed a computational model for predicting tiredness during exercise from a sitting to a standing posture, which could be useful for rehabilitation. Si et al. [36] employed the SVM and fractal analysis for gait recognition and test the identification performance, and the testing outcomes indicate an overall accuracy of 93.57% via radial basis function kernel. Jeong et al. [37] used the SVM to classify activity patterns based on plantar pressure characteristics, and the recognition rate reached 95.2%. Stetter et al. [38] used the SVM and identified the kinematic difference between fatigue and nonfatigue based on principal component analysis, the strides of fatigue and nonfatigue were separated, and the classification accuracy was 99.4%. Wang et al. [39] used inertial measurement unit (IMU) and SVM to distinguish fatigue and nonfatigue running states, and predict the degree of fatigue. The classification accuracy of tibia and thigh IMUs was 91.10%. The characteristics of plantar pressure were evaluated using leave-one-out cross-validation with machine learning algorithms: SVM, decision tree, discriminant analysis, and k-nearest neighbors in the study of Merry et al. [17]. The results showed that the SVM and decision tree have higher classification accuracy. In addition, other studies have shown that the SVM has the best performance in distinguishing gait characteristics [40]. Therefore, the SVM was used to predict fatigue in this study. In addition, many researchers have applied SVM to the recognition of gait characteristics before and after fatigue, but few studies have paid attention to the plantar mechanical characteristics before and after fatigue.

As a consequence, this research aimed to explore the differences in peak pressure (PP), peak force (PF), and plantar impulse (PI) before and after long-distance running fatigue in novice runners, as well as gender differences. We also employed the SVM algorithm to predict fatigue based on plantar mechanical parameters. Based on previous studies, we assumed that the change in plantar mechanical parameters before and after fatigue mainly occurred in the toes. It was also assumed that gender differences in plantar mechanical parameters were mainly concentrated in the toes and metatarsal regions. In addition, it was assumed that the SVM can predict fatigue at a high level.

## 2. Materials and Methods

### 2.1. Participants

The experimental subjects for this investigation were 15 healthy males and 15 healthy females [13, 25] who were novice runners (Table 1) with dominant right legs. Participants were recruited from sports clubs at Ningbo University and via social media. There were no health issues, neuromuscular abnormalities, or recognized gait difficulties in any of the participants, and no lower limb injuries in six months before the experiment. High arches and flat feet were not allowed to participate in the recruitment process. All subjects were given and signed written consent granted by the Institutional Review Board before the experiment (RAGH20210922205.6).

### 2.2. Experimental Procedures

**Figure 1:**
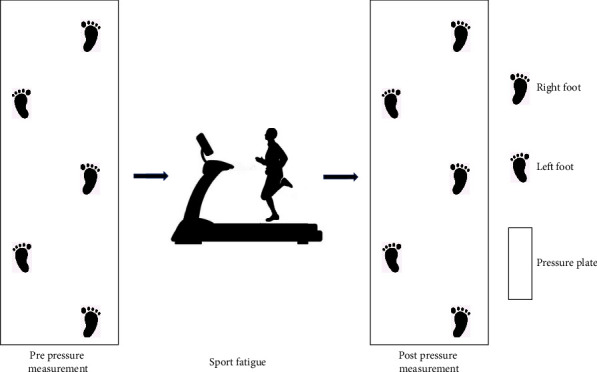
The experimental procedure.

Figure 1 depicts the experimental procedure. All of the participants did fatigue-inducing running workouts. The 15-point Borg scale and heart rate monitor (Polar RS100, Polar Electro Oy, Woodbury, NY, USA) were used to record perceived exertion, and heart rate changes per minute during the fatigue intervention. The individuals began the experiment by running at a speed of 1.67 m/s on a treadmill (h/p/cosmos para graphics^R^, Germany). During the experiment, the slope was maintained at 1% [41–43]. After which the speed was increased by 0.28 m/s every 2 minutes until the subjects reached a Borg intensity of 13. The subjects then continued at this speed until they reached Borg scale 17 or 90% of maximal heart rate (HRmax calculated at 220-age), at which point they slowly reduced the speed to a speed of their choice [44, 45]. Space constraints, repeatability, and better control of climate, speed, and slope were the reasons why treadmill running was selected by our research team [46].

In this experiment, a footscan pressure plate (Footscan® software 7.0 Gait 2nd Generation, RsScan International) was used to monitor dynamic plantar pressure. The footscan pressure plate was 2 m in length and the acquisition frequency was 126 Hz. Subjects were asked to perform a pressure measurement on the footscan pressure plate before and immediately after fatigue. To avoid injury during the test, the subjects familiarized themselves with the footscan pressure plate before the trial. After familiarity, the subjects were asked to run on the footscan pressure plate at a speed of 3.3 m/s ± 5% [44]. To manage running speed, Brower timing lights (Brower Timing System, Draper, UT, USA) were used. The subjects who completed a full gait cycle on the footscan pressure plate at the specified speed were regarded as successful. 5 groups of valid data were collected from each subject before and after the fatigue intervention. In addition, during the fatigue intervention, we uniformly provided clothes and shoes to the subjects to avoid experimental differences and maintain consistency.

### 2.3. Data Analysis

**Figure 2:**
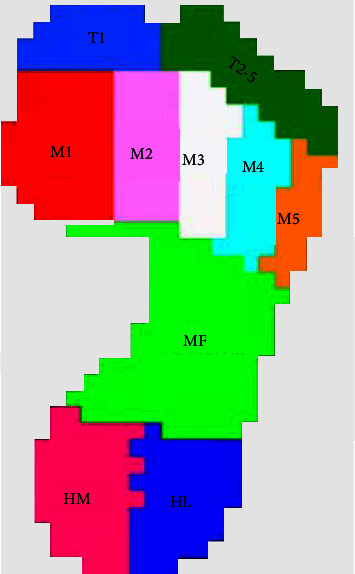
The location of ten anatomical zones on the peak mechanical footprint.

We analyzed plantar mechanical parameters in the running stance phase. For each trial, ten anatomical zones were automatically identified by the software (Footscan® software 7.0 Gait 2nd Generation, RsScan International) and if necessary, manually corrected by adjusting the pixels per zone (Figure 2): hallux *(T*1), second-fifth toes (*T*2–5), metatarsal 1–5 (*M*1, *M*2, *M*3, *M*4, *M*5), midfoot (MF), heel medial (HM), and heel lateral (HL). During the adjustment, we performed strict controls to ensure that the adjustment conditions and adjustment levels were rigorous and careful. The average values of PP, PF, and PI for all ten regions were calculated.

### 2.4. Statistical Analysis and SVM Classification Algorithm

The calculated data were exported to a statistical software package SPSS 26.0 (SPSS, Chicago, IL, USA), and the peak pressure, peak force, and plantar impulse of each plantar zone before and after running were statistically processed. The data were initially assessed for normality using a Kolmogorov–Smirnov test. The data were normally distributed. To investigate the effects of fatigue, gender, and their interaction on the plantar mechanical parameters, a two-way analysis of covariance (ANCOVA) was conducted. The significance level was set as *P* < 0.05.

When the data sets were not easily separable, the SVM classifier is a supervised machine learning technique that translates the input data space to a higher dimensional space to obtain a more accurate classification [35]. In our study, we used the LIBSVM toolbox based on MATLAB 2016b (Mathworks, MA, USA). The linear kernel was used for the SVM in the study. The cross-validation technique we employed was the hold-out method. 66.7% of the sample size was randomly selected as the training set, and 33.3% of the sample size was used as the test set [41].

The SVM is suitable for small and medium data samples, and nonlinear, high-dimensional classification problems. It maps the feature vector of the instance to some points in space. The purpose of the SVM is to find a line that best distinguishes two types of points, and when new points are added later, this line can also make a good classification. The SVM will find the partitioning hyperplane that distinguishes the two classes and maximizes the separation. For any hyperplane, the data points on both sides have a minimum distance (vertical distance) from it, and the sum of these two minimum distances is the interval.

For this partitioned hyperplane, we can give the following equation:

(1) $$\begin{array}{c} \omega^{T}X+b=0\text{,} \end{array}$$

where *ω* is the weight of each feature and the column vector. *b* is the displacement value.

The distance from the point *x*_*i*_ to the surface is as follows:

(2) $$\begin{array}{c} \frac{\omega^{T}x_{i}+b}{\left\|\omega \right\|}\text{.} \end{array}$$

Then,

(3) $$\begin{array}{c} \frac{\omega^{T}x_{i}+b}{\left\|\omega \right\|}\times y_{i}\geq d\text{,} \end{array}$$

*y*_*i*_ is the predicted value of sample *i* (−1 or 1, doing sign transformation). *d* is the distance of the support vector to the hyperplane. We assume that *d* is (2/‖*ω*‖).

Making all the points meet:

(4) $$\begin{array}{c} y_{i}\left(\omega^{T}x_{i}+b\right)\geq 1\text{.} \end{array}$$

The hyperplane we need is the one that needs to maximize the minimum interval, i.e.,

(5) $$\begin{array}{c} \underset{\omega ,b}{\mathrm{argmax}}\left\{\frac{1}{\left\|\omega \right\|}\min_{i}\left[y_{i}\left(\omega^{T}∙x_{i}+b\right)\right]\right\}\text{.} \end{array}$$

Then, we need to calculate

(6) $$\begin{array}{c} \underset{\omega ,b}{\mathrm{argmax}}\frac{1}{\left\|\omega \right\|}\text{.} \end{array}$$

Equivalent to calculate

(7) $$\begin{array}{c} \min_{\omega ,b}\frac{1}{2}{\left\|\omega \right\|}^{2}\text{,} \end{array}$$

and

(8) $$\begin{array}{c} y_{i}\left(\omega^{T}x_{i}+b\right)\geq 1\text{.} \end{array}$$

Using the Lagrange multiplier method:

(9) $$\begin{array}{c} =\frac{1}{2}{\left\|\omega \right\|}^{2}-\overset{n}{\underset{i=1}{\sum }}a_{i}\left(y_{i}\left(\omega^{T}x_{i}+b\right)-1\right.\text{.} \end{array}$$

The original problem is the minimax problem.

(10) $$\begin{array}{c} \min_{\omega ,b}\max_{\alpha }L\left(\omega ,b,\alpha \right)\text{.} \end{array}$$

The dual problem of the original problem is a maximin problem:

(11) $$\begin{array}{c} \max_{\omega ,b}\min_{\alpha }L\left(\omega ,b,\alpha \right)\text{.} \end{array}$$

Taking its partial derivative with respect to *ω* and *b* and making it equal to 0,

(12) $$\begin{array}{c} \omega =\overset{n}{\underset{i=1}{\sum }}\alpha_{i}y_{i}x_{n}\text{,}\\ \overset{n}{\underset{i=1}{\sum }}\alpha_{i}y_{i}=0\text{.} \end{array}$$

Then,

(13) $$\begin{array}{c} L\left(\omega ,b,\alpha \right)=\overset{n}{\underset{i=1}{\sum }}\alpha_{i}-\frac{1}{2}\overset{n}{\underset{i=1}{\sum }}\alpha_{i}\alpha_{j}y_{i}y_{j}x_{i}x_{j}\text{.} \end{array}$$

Combining the abovementioned condition:

(14) $$\begin{array}{c} \begin{array}{c} \overset{n}{\underset{i=1}{\sum }}\alpha_{i}y_{i}=0\\ \alpha_{i}\geq 0,i=1,2\cdots n \end{array}\text{.} \end{array}$$

Then, we find the maximum value of *α*.

(15) $$\begin{array}{c} \min\frac{1}{2}\overset{n}{\underset{i=1}{\sum }}\alpha_{i}\alpha_{j}y_{i}y_{j}x_{i}x_{j}-\overset{n}{\underset{i=1}{\sum }}\alpha_{i}\text{.} \end{array}$$

We continue to use Lagrange multipliers:

(16) $$\begin{array}{c} \omega^{*}=\overset{N}{\underset{i=1}{\sum }}a_{i}^{*}y_{i}x_{i}\text{,}\\ b^{*}=y_{i}-\overset{N}{\underset{i=1}{\sum }}a_{i}^{*}y_{i}x_{i}x_{j}\text{.} \end{array}$$

We find the final hyperplane.

## 3. Results

### 3.1. The Peak Pressure

**Figure 3:**
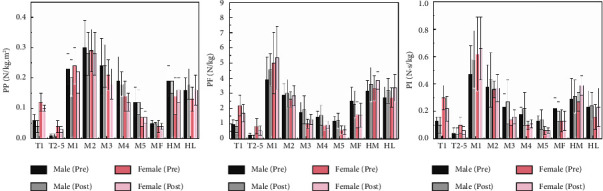
Description of PP, PF, and PI by gender before and after running fatigue.

According to Figure 3 and Table 2, fatigue mainly affected the PP at *T*1, *T*2–5, HM, and HL, and gender factors were mainly reflected at *T*1, *T*2–5, and *M*3–5. Specifically, PP decreased significantly in *T*1 and *T*2–5 regions after fatigue (*P* < 0.05) and increased significantly in HM and HL (*P* < 0.05). PP at *T*1 and *T*2–5 was significantly higher in females than in males (*P* < 0.05), and PP at *M*3–5 was significantly higher in males than in females (*P* < 0.05).

### 3.2. The Peak Force

According to Figure 3 and Table 2, the fatigue effect was mainly reflected in the *T*1, *T*2–5, *M*1, HM, and HL, while the gender effect was mainly reflected in *T*1, *T*2–5, and *M*3–5. PF was significantly decreased at *T*1 and *T*2–5 due to fatigue and significantly increased at M1, HM, and HL (*P* < 0.05). PF in females was significantly larger at *T*1 and *T*2–5 than that in males, and significantly smaller at *M*3–5 than that in males (*P* < 0.05).

### 3.3. The Impulse

According to Figure 3 and Table 2, the fatigue effect was mainly reflected in the toes, *M*1, and heel, while the gender effect was mainly reflected in *T*1, *T*2–5, and *M*3–5. PI decreased significantly at *T*1 and *T*2–5 after fatigue (*P* < 0.05), and increased significantly at M1, HM, and HL (*P* < 0.05). In addition, the PI at *T*1 and *T*2–5 showed that females were significantly larger than males, and at *M*3–5, females were significantly smaller than males (*P* < 0.05).

### 3.4. SVM Classification Algorithm

**Figure 4:**
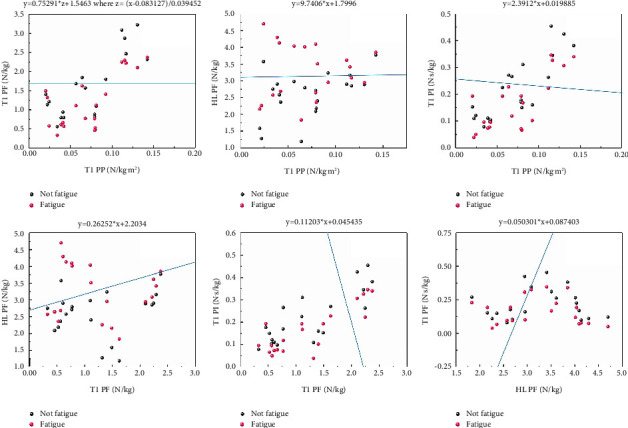
SVM classification algorithm results.

We selected combinations of plantar zone parameters with significant differences (*P* < 0.001.  Figure 4 shows the best fit separating hyperplane lines of fatigue or not fatigue in different plantar zone parameter combinations. The accuracy of the different plantar zone parameter combinations in predicting fatigue is presented in Table 3. The results showed that the average accuracy was a moderate level (train accuracy: 62.5%; test accuracy: 62.5%). The accuracies of following combinations were above average and showed a high level: *T*1 PP/HL PF (train accuracy: 65%; test accuracy: 75%), *T*1 PF/HL PF (train accuracy: 67.5%; test accuracy: 65%), and HL PF/*T*1 PI (train accuracy: 67.5%; test accuracy: 70%).

## 4. Discussion

This research aimed to analyze how PP, PF, and PI changed before and after running fatigue in novice runners, as well as gender differences. Based on previous studies, we assumed that the changes in plantar mechanical parameters before and after fatigue mainly occurred in *T*1 and *T*2–5. It was also assumed that gender differences in plantar parameters were mainly concentrated in *T*1 and *T*2–5 and *M*1–5. In addition, it was assumed that SVM can predict fatigue at a high level. Our results are largely consistent with our previous assumptions.

The changes in plantar mechanical parameters caused by fatigue were mainly under the *T*1, *T*2–5, *M*1, HM, and HL. The plantar mechanical parameters in the toes region were also reduced in the research of Bisiaux and Moretto [13], Karagounis et al. [47], and Willems et al. [48]. This may be due to the increased dorsiflexion of the metatarsophalangeal joint after fatigue, which leads to fewer toes contributing to running, and thus less load under the toes [49]. In a study of PP and center of pressure (COP), it was found that the PP under the toes decreased, with a retraction of the COP. According to Stolwijk et al. [50], to avoid overuse of the forefoot and the risk of incurring forefoot pain, subjects adjusted their gait pattern. This could explain why plantar mechanical parameters under the toes were decreased. Nagel et al. [49] also noted a decline in toes load. However, in the study of Bisiaux and Moretto [13] and Weist et al. [51], the phenomenon of decreased load under the toes was not observed. Moreover, in the study of Willems et al. and Wu et al. [48, 52], it was also found that PF and PI at *M*1 increased significantly after fatigue, which was consistent with our findings. Perhaps because of the reduction in mechanical parameters under the toes, the load was transferred to the metatarsal. However, increased submetatarsal load may contribute to the occurrence of metatarsal stress fractures [49]. An investigation also revealed that after running fatigue, the contact area of the HM grew while the HL reduced. Then led to greater pronation in the rearfoot [53]. If fatigued, the quadriceps need to play a greater role to avoid knee instability, resulting in less knee flexion, which leads to increased heel pressure. This explanation has also been confirmed by Stolwijk et al. [50].

Several gender-induced differences in plantar mechanical parameters were found. PP, PF, and PI were significantly higher in females than in males at *T*1 and *T*2–5 and significantly higher in males than in females at *M*3–5. This was also reflected in studies of Ferrari et al. [54] and Demirbuken et al. [27]. The larger plantar mechanical parameters of females' *T*1 and *T*2–5 may be related to the fact that females wear high heels, which also raised the risk of chronic paraspinal muscle fatigue, which was linked to postural changes and pain [55]. Although this study did not include cases of hallux valgus (HV), females had a higher load of the hallux than males. Studies reported a meta-analysis that estimated that female HV prevalence (30%) was 2.3 times greater than that in males (13%) [21]. Although many studies cannot reach a unified conclusion, there was no denying that gender differences in plantar mechanical parameters may be one of the reasons for the increase in hallux valgus in females. Males had much more load in the forefoot area than females, which could be due to males' higher body weight, physical structural differences, and females' better flexibility [56, 57]. Further to this, males tend to have a higher vertical center of mass displacement during walking than females. This may also contribute to a higher load in *M*3–5 [56]. Pressure is equal to force divided by area. In all regions of the foot, males had a considerably higher contact area than females, both statistically and clinically [25]. At the same time, because of the female hormone secretion, the foot ligament relaxation reduces the degree of stiffness and spreads the forces to a larger extent [58, 59]. This may be the reason that the PP, PF, and PI at *M*3–5 are higher in males than in females to varying degrees. In this study, gender differences in PP, PF, and PI were mainly found in the *T*1, *T*2–5, and *M*3–5. Although the literature's findings are not always consistent, factors including gender and foot anatomy are thought to be linked to metatarsal stress fractures and lower limb injuries [50].

During running, the feet are the only part of the body that makes direct touch with the ground, and they are critical to progress. Running actions may be hampered by muscle exhaustion and physical discomfort. As a result, it is theoretically possible to predict fatigue through plantar mechanical parameters. Previous research has shown that fatigue will affect plantar mechanical parameter distribution and fatigue is correlated with plantar mechanical parameters [48]. Interval maximization is the SVM classification algorithm, which may be characterized as a problem of solving convex quadratic programming and is equivalent to the regularized hinges loss work minimization issue. The SVM classification algorithm is an optimization algorithm for solving convex quadratic programming, as evidenced by our SVM classification algorithm results. The SVM classification algorithm results revealed that the mean accuracy was an above-moderate level. The accuracy was of an above-average level by using the T1 PP/HL PF, T1 PF/HL PF, and HL PF/T1 PI. These indicated that fatigue can be predicted to a certain extent by monitoring plantar mechanical parameters before and after running fatigue. Running fatigue can be predicted using the learned SVM classification algorithm, which can also be used as a useful tool for fatigue supervision. The learned SVM classification algorithm can help coaches to better identify the physical state of athletes from start to the finish of a run by monitoring plantar mechanical parameters. The classification may also be useful in identifying injuries over the running season.

There are some limits of this study. In the experiment, a treadmill was used for the fatigue intervention. We only studied the plantar mechanical parameters under treadmill conditions but did not study the condition of running on the ground. Future studies should include subjects performing at different exercise levels, such as professional athletes. In addition, the sample size should be expanded to improve the accuracy of the SVM classification algorithm.

## 5. Conclusions

We found that the change of plantar mechanical parameters caused by fatigue was mainly concentrated in *T*1, *T*2–5, M1, HM, and HL. While the effect of gender was mainly found in the *T*1, *T*2–5, and *M*3–5. These may indicate injuries related to fatigue and gender factors, such as metatarsal stress fractures and HV. Plantar mechanical parameters can be monitored before and after long-distance running to predict fatigue to some extent. The learned algorithm of plantar zone combinations with above-average accuracy (T1 PP/HL PF, T1 PF/HL PF, and HL PF/T1 PI) can predict long-distance running fatigue and provide supervised training strategies.

## Figures and Tables

**Table 1:** Demographic data.

	Age (years)	Height (m)	Body mass (kg)	BMI (kg/m^2^)	Shoe size (cm)
Male	23.61 (0.92)	1.83 (0.12)	76.14 (8.12)	24.87 (2.32)	26.35 (0.49)
Female	23.07 (1.04)	1.69 (0.13)	54.73 (4.14)	20.13 (1.08)	24.22 (0.34)

^*∗*^Values: mean (SD).

**Table 2:** Changes in PP, PF, and PI before and after fatigue.

	Male/pre	Male/post	Female/pre	Female/post	*P* value		
					*F*	*G*	*F* × *G*
PP (N/kg·m^2^)							
*T*1	0.06 (0.02)	0.04 (0.02)	0.12 (0.03)	0.10 (0.01)	**0.001**	**<0.00**1	0.693
*T*2–5	0.01 (0.005)	0.01 (0.005)	0.04 (0.02)	0.03 (0.01)	**0.002**	**<0.001**	0.115
*M*1	0.23 (0.05)	0.20 (0.06)	0.24 (0.06)	0.22 (0.07)	0.583	0.396	0.056
*M*2	0.30 (0.09)	0.28 (0.07)	0.29 (0.07)	0.28 (0.07)	0.256	0.99	0.476
*M*3	0.24 (0.09)	0.24 (0.07)	0.21 (0.05)	0.18 (0.05)	0.423	**0.031**	0.156
*M*4	0.19 (0.08)	0.18 (0.04)	0.14 (0.05)	0.12 (0.03)	0.238	**<0.001**	0.842
*M*5	0.12 (0.05)	0.12 (0.04)	0.07 (0.03)	0.07 (0.02)	0.832	**<0.001**	0.983
MF	0.05 (0.01)	0.05 (0.003)	0.04 (0.02)	0.04 (0.01)	0.283	0.067	0.086
HM	0.19 (0.05)	0.19 (0.04)	0.14 (0.06)	0.16 (0.04)	**0.034**	0.16	0.453
HL	0.16 (0.04)	0.18 (0.05)	0.13 (0.04)	0.16 (0.05)	**0.007**	0.29	0.449
PF (N/kg)							
*T*1	0.96 (0.29)	0.81 (0.40)	2.20 (0.67)	1.69 (0.58)	**<0.001**	**0.001**	0.063
*T*2–5	0.25 (0.14)	0.13 (0.11)	0.83 (0.50)	0.54 (0.30)	**0.003**	**0.004**	0.098
*M*1	3.92 (1.46)	4.54 (1.07)	5.00 (2.03)	5.34 (2.09)	**0.017**	0.213	0.309
*M*2	2.87 (0.75)	3.00 (0.92)	2.60 (0.49)	2.83 (0.66)	0.163	0.235	0.975
*M*3	1.76 (0.67)	1.91 (0.92)	0.99 (0.30)	1.27 (0.33)	0.579	**0.014**	0.084
*M*4	1.45 (0.38)	1.55 (0.66)	0.88 (0.30)	0.92 (0.26)	0.578	**0.001**	0.334
*M*5	1.18 (0.31)	1.20 (0.50)	0.58 (0.27)	0.59 (0.29)	0.968	**<0.001**	0.936
MF	2.50 (0.90)	2.27 (0.86)	1.59 (0.87)	1.55 (0.80)	0.079	0.055	0.097
HM	3.14 (0.71)	3.59 (1.12)	3.34 (0.82)	3.85 (0.57)	**0.005**	0.483	0.838
HL	2.70 (0.73)	3.18 (0.81)	2.73 (0.64)	3.36 (0.89)	**<0.001**	0.702	0.285
PI (N·s/kg)							
*T*1	0.13 (0.03)	0.10 (0.06)	0.30 (0.09)	0.22 (0.09)	**<0.001**	**<0.001**	0.072
*T*2–5	0.04 (0.04)	0.03 (0.05)	0.10 (0.06)	0.06 (0.03)	**0.017**	**0.001**	0.078
*M*1	0.47 (0.21)	0.58 (0.21)	0.62 (0.27)	0.66 (0.23)	**0.022**	0.405	0.054
*M*2	0.38 (0.16)	0.43 (0.20)	0.36 (0.06)	0.37 (0.10)	0.296	0.162	0.234
*M*3	0.23 (0.10)	0.27 (0.16)	0.14 (0.04)	0.16 (0.05)	0.901	**0.006**	0.109
*M*4	0.18 (0.05)	0.22 (0.12)	0.10 (0.03)	0.11 (0.03)	0.369	**<0.001**	0.499
*M*5	0.13 (0.04)	0.14 (0.07)	0.06 (0.03)	0.06 (0.02)	0.597	**<0.001**	0.711
MF	0.22 (0.08)	0.20 (0.07)	0.13 (0.08)	0.13 (0.07)	0.243	0.066	0.185
HM	0.29 (0.15)	0.31 (0.12)	0.27 (0.07)	0.39 (0.07)	**0.021**	0.478	0.639
HL	0.23 (0.12)	0.24 (0.10)	0.16 (0.09)	0.23 (0.14)	**0.011**	0.386	0.413

Pre = before fatigue, post = after fatigue, *F* = fatigue, and *G* = gender. The values in bold in the table show significant differences; *P* < 0.05; values: mean (SD). The bold data in the table indicate statistical significance.

**Table 3:** The accuracy of the SVM classification algorithm results.

Plantar zone parameters	Train accuracy (%)	Test accuracy (%)
T1 PP/T1 PF	55	55
T1 PP/HL PF	**65**	**75**
T1 PP/T1 PI	65	55
T1 PF/HL PF	**67.5**	**65**
T1 PF/T1 PI	55	55
HL PF/T1 PI	**67.5**	**70**
Mean accuracy	62.5	62.5

The data in bold indicates that train accuracy and test accuracy are above mean accuracy concurrently.

## Data Availability

The data used to support the findings of this study are available from the corresponding authors upon request.
